# BGGE: A New Package for Genomic-Enabled Prediction Incorporating Genotype × Environment Interaction Models

**DOI:** 10.1534/g3.118.200435

**Published:** 2018-07-26

**Authors:** Italo Granato, Jaime Cuevas, Francisco Luna-Vázquez, Jose Crossa, Osval Montesinos-López, Juan Burgueño, Roberto Fritsche-Neto

**Affiliations:** * Department of Genetics, “Luiz de Queiroz” College of Agriculture, University of São Paulo, Piracicaba, São Paulo, Brazil; † Universidad de Quintana Roo, Chetumal, Quintana Roo, México; ‡ Biometrics and Statistics Unit, International Maize and Wheat Improvement Center (CIMMYT), Apdo. Postal 6-641, 06600, México DF, México; § Facultad de Telemática, Universidad de Colima, Colima, Colima, 28040, México,

**Keywords:** GE: genotype × environment (GE), BGGE: Bayesian Genomic Genotype × Environment Interaction, GS: Genomic Selection, BGLR: Bayesian Genomic Linear Regression, GenPred, Shared Data Resources

## Abstract

One of the major issues in plant breeding is the occurrence of genotype × environment (GE) interaction. Several models have been created to understand this phenomenon and explore it. In the genomic era, several models were employed to improve selection by using markers and account for GE interaction simultaneously. Some of these models use special genetic covariance matrices. In addition, the scale of multi-environment trials is getting larger, and this increases the computational challenges. In this context, we propose an R package that, in general, allows building GE genomic covariance matrices and fitting linear mixed models, in particular, to a few genomic GE models. Here we propose two functions: one to prepare the genomic kernels accounting for the genomic GE and another to perform genomic prediction using a Bayesian linear mixed model. A specific treatment is given for sparse covariance matrices, in particular, to block diagonal matrices that are present in some GE models in order to decrease the computational demand. In empirical comparisons with Bayesian Genomic Linear Regression (BGLR), accuracies and the mean squared error were similar; however, the computational time was up to five times lower than when using the classic approach. Bayesian Genomic Genotype × Environment Interaction (BGGE) is a fast, efficient option for creating genomic GE kernels and making genomic predictions.

Genomic selection has the advantage of saving time and resources when selecting genotypes by employing genomic-enabled prediction methods for complex traits, along with pedigree information, molecular markers and/or environmental covariates (Crossa *et al.* 2017). In the genomic selection method proposed by Meuwissen *et al.* (2001), Bayesian models were introduced in the context of whole-genome regression; they have become common in genomic prediction (Gianola 2013). Within this framework, appropriate prior distributions and simulations via Markov Chain Monte Carlo (MCMC) allow convergence for predictive posterior distributions that cannot be solved analytically. However, these methods require thousands of iterations to ensure convergence, so that if the model is complex, the sampling process can increase the computational time. In this context, attempts have been made to reduce the computational time of Bayesian models with approaches that do not use MCMC, such as variational Bayesian methods (Montesinos-López *et al.* 2017) and Integrated Nested Laplace Approximation (INLA) (Holand *et al.* 2013; Mathew *et al.* 2016). These methods are faster; however, they have constraints that may lead to lower prediction accuracy, which is undesired.

Using molecular markers (*p*) in classic parametric regression with n individuals can lead to the problem of n≪p setting, which can be reduced by using semi-parametric regression such as Reproducing Kernel Hilbert Spaces (RKHS) (Gianola and Van Kaam 2008; de los Campos *et al.* 2010). These approaches assume the contribution of molecular markers as a random variable in some distributions with a covariance matrix that consists of a scalar variance component and a known covariance kernel obtained by markers. This covariance kernel can model genetic effects as additive, dominance and epistasis, as a mixture of these effects or even as genetic and non-genetic remaining effects (Crossa *et al.* 2010; Technow *et al.* 2012; Azevedo *et al.* 2015). Genomic-enabled predictions are usually done using models that do not take into account genotype × environment interaction (GE). Nevertheless, the advantage of genomic models that take into account information from multi-environment trials simultaneously has been proved (Burgueño *et al.* 2012). Hence, a family of genomic models was developed to account for GE interaction; these models also allow incorporating fixed effects of environments and several genetic and environmental effects into a variety of linear mixed models (Jarquín *et al.* 2014; Lopez-Cruz *et al.* 2015; Sousa *et al.* 2017).

In this paper, we describe the Bayesian Genomic Genotype × Environment (BGGE) R package that fits genomic linear mixed models to single environments and multi-environments with GE models. The increase in speed is achieved by reparametrization through orthogonal rotation of the random vectors, allowing the use of univariate distributions in the sampling process (Cavalier 2008; Cuevas *et al.* 2014). Also, some special treatments are given for structured dispersed covariance matrices, in particular, those structured as a block diagonal, prevalent in some GE models (Sousa *et al.* 2017). We present statistical models and algorithms with a generic linear mixed model and its Bayesian counterpart, which is the base of the BGGE package, as well as the most representative part of the prediction process and kernel construction for genomic models. In addition we describe the *getK* and *BGGE* functions, which offer the possibility of fitting six different multi-environment genomic models with GE based on models proposed by Jarquín *et al.* (2014) and Lopez-Cruz *et al.* (2015),; we also give some examples of their use, and compare them to other packages that use Bayesian approaches.

We note that although the *getK* function is an auxiliary function, it allows fitting not only the six genomic multi-environment models with GE, but can also model several other situations. However, the potential of the package is given by the *BGGE* function that provides the versatility needed to fit a great number of different genomic data sets.

## Statistical Models

### Linear mixed model

Consider the following basic linear mixed models that cover the diversity of models that can be applied to single or multi-environment trials. Assume q vectors of random effects:

$$y=\mu 1+X_{f}\beta +\overset{q}{\underset{r=1}{\sum }}u_{r}+\varepsilon$$ (1)

where y is the vector combining the genotypic means of observations. The scalar μ is the common intercept or the mean. Matrix $X_{f}\;$represents the design matrix associated with the vector of fixed effects β. Random vectors $u_{r}$ (r=1, 2,…,q) are assumed to be independent of other random effects. We expected that $u_{r}$ would follow a normal distribution with zero mean and a covariance matrix of the form ${\sigma^{2}}_{u_{r}}K_{r}$, where ${\sigma^{2}}_{u_{r}}$ is a scalar representing the unknown variance parameter to be estimated from $u_{r}$, and $K_{r}$ is a known symmetric positive semi-definite covariance matrix. Model (1) is very general and it can be used to model different problems in biology or other areas, particularly genomic selection areas. It should be pointed out that in this first version of the BGGE package, the design matrix $X_{f}$ is limited to being a full rank matrix with the vectors $u_{r}$ being of the same size as y, and representing in the most common case, a reparameterization equivalent to $Z_{u}u$ or $Z_{g}g$ used in mixed models for genomic selection (Crossa *et al.* 2010, Jarquín *et al.* 2014), where $Z_{u}\;$or $Z_{g}$ are known incidence matrices that relate the genotypes to the observations, and g or **u** are the random genetic effects of the genotypes (a known matrix multiplied by a random vector results in a random vector of the same size as the response variable vector y).

Finally, random error vector ε, of the same length as y, follows a normal distribution with zero mean and form ε∼ N(0,Σ), where Σ is a covariance matrix $\Sigma =I\sigma_{\varepsilon }^{2}$, and I is an identity matrix. The previous assumptions allow the BGGE models to be used only with continuous data assumed to have a multivariate normal distribution with observations (not independent) that depend on the variance-covariance structure of the genotypes. The main objective of the BGGE is to focus on the covariance structure more than on the possible heteroscedasticity/homoscedasticity of the error.

### Linear mixed model parametrization

The main objective of the reparameterization of model (1) is to rotate the dependent observations in the response vector y that follows a multivariate normal distribution to an orthogonal space that ensures independence. This rotation allows overcoming matrix problems (*e.g.*, not full rank matrices), thus vectorising matrices that result in much faster computation and estimation of the model’s parameters. This rotation is achieved with the decomposition or factorization of matrices such as singular value decomposition (SVD) or eigen-decomposition that are commonly used in parametric regression models like principal component regression or in genomic-enabled prediction (Cuevas *et al.* 2014, Meuwissen *et al.* 2017).

In linear mixed models, the covariance matrix is symmetric and positive semidefinite and can be factorized by using the eigen-decomposition of K of order n × *n* (de los Campos *et al.* 2010). Hence, K=USU', where S is a diagonal matrix with n non-zero eigenvalues and U is an orthogonal matrix with eigenvectors associated with n eigenvalues. To facilitate reading, we use a single kernel model, considering that $y^{*}=y-\mu 1-X_{f}\beta$. Cuevas *et al.* (2014) proposed an orthogonal transformation by multiplying both sides of (1) by U':

$$y^{*}=u+\varepsilon$$

$$\mathrm{U'}y^{*}=\mathrm{U'}u+{\mathrm{U'}}^{'}\varepsilon$$ (2)

such that model (2) becomes:

d=b+e  (3)

where $d={\mathrm{U'}}^{'}y^{*}$ b=U'u and$\;e={\mathrm{U'}}^{'}\varepsilon$. The model assumes that b comes from a normal distribution such that $\mathrm{U'}u∼N\left(0,{\mathrm{U'}}^{'}\mathrm{KU}\sigma_{u}^{2}\right)=N\left(0,\mathrm{U'}\mathrm{US}\mathrm{U'}U\sigma_{u}^{2}\right)=N(0,S\sigma_{u}^{2})$, considering that U'U=I. Similarly, model (2) assumes that e comes from a normal distribution such that ${\mathrm{U'}}^{'}\varepsilon ∼N\left(0,\mathrm{U'}U\sigma_{\varepsilon }^{2}\right)=N(0,I\sigma_{\varepsilon }^{2})$. The rotation causes the elements of b to be independent with univariate normal distributions. It is also worth noting that eigenvalues that are very close to zero (less than $1\times 10^{-10}$) reflect the noise (and numerical errors) and their associated eigenvectors can be eliminated, thereby reducing the dimension of matrices U and S. Note that the proposed matrices K were previously scaled in order to reduce their magnitude. In addition, the BGGE package offers an argument (tol = tolerance) to change the default value of ($1\times 10^{-10}$) for the eigenvalues considered equal to zero.

### Bayesian linear mixed models

The BGGE solves the linear mixed models through Bayesian hierarchical modeling. The distribution of the transformed data d, given b and $\sigma_{\varepsilon }^{2}$, is:

$$f\left(d|b,\sigma_{\varepsilon }^{2}\right)=\overset{n}{\underset{i=1}{\prod }}N\left(d_{i}|b_{i},\sigma_{\varepsilon }^{2}\right)$$ (4)

The Bayesian linear mixed model assumes that $p\left(u|\sigma_{u}^{2}\right)=N\left(u|0,K\sigma_{u}^{2}\right)$; then the conditional distribution of $b_{i}$ is as follows:

$$p\left(b_{i}|\sigma_{u}^{2}\right)=N\left(b_{i}|0,\sigma_{u}^{2}s_{i}\right)$$ (5)

where $s_{i}$ are the eigenvalues and $\sigma_{u}^{2}\;$is the unknown scale. This reparameterization allows sampling from univariate normal distributions, making the convergence process simpler and faster.

The proposed conjugate prior distribution for $\sigma_{u}^{2}$ is a scaled inverse chi squared, p($\sigma_{u}^{2})∼\chi^{-2}\left(\nu_{u},Sc_{u}\right)$, where $\nu_{u}$ denotes the degree of freedom and $Sc_{u}$ represents the scale factor. In the BGGE package, the degrees of freedom are set to a value of 3 with the idea of ​​not generating infinite values in the samples of $\sigma_{u}^{2}$. On the other hand, the prior distribution used for $Sc_{u}$ was previously computed from the data, as suggested by Pérez and de los Campos (2014) (see details in the Appendix). The conjugate prior distribution used for $\sigma_{\varepsilon }^{2}$ is a scaled inverse chi squared, p($\sigma_{\varepsilon }^{2})∼\chi^{-2}\left(\nu_{\varepsilon },Sc_{\varepsilon }\right)$, where $\nu_{\varepsilon }$ represents the degrees of freedom and $Sc_{\varepsilon }$ denotes the scale factor.

Hence, the joint posterior distribution of $\left(b,\;\sigma_{u}^{2},\sigma_{\varepsilon }^{2}\right),$ given d$,\nu_{u},Sc_{u},\nu_{\varepsilon },Sc_{\varepsilon }\text{and}S$, is:

$$p\left(b,\sigma_{u}^{2},\;\sigma_{\varepsilon }^{2}|d,\nu_{u},Sc_{u},\nu_{\varepsilon },Sc_{\varepsilon },S\right)\propto \left\{\overset{n}{\underset{i=1}{\prod }}N\left(d_{i}|b_{i},\sigma_{\varepsilon }^{2}\right)N\left(b_{i}|0,\sigma_{u}^{2}s_{i}\right)\right\}\times \chi^{-2}\left(\sigma_{u}^{2}|\nu_{u},\nu_{u}Sc_{u}\right)\times \chi^{-2}\left(\sigma_{\varepsilon }^{2}|\nu_{\varepsilon },\nu_{\varepsilon }Sc_{\varepsilon }\right)$$ (6)

From equations (5) and (6), conditional distributions can be constructed to generate the MCMCs through a Gibbs sampler. Note that u genetic values can be recovered from u=Ub. Details are presented in the Appendix.

### Sparse matrices

In an attempt to speed up the prediction algorithm, several special treatments are given for sparse matrices. In several GE models (Jarquín *et al.* 2014; Lopez-Cruz *et al.* 2015), some random u effects have an associated covariance matrix that can be considered sparse with submatrices in a known structure. Thus, instead of applying eigen-decomposition in the complete matrix, we identify, individualize and apply eigen-decomposition in the submatrices that compose the block diagonal; this speeds up eigen-decomposition and makes the multiplication of matrices and vectors faster, thus reducing the iteration time.

### Obtaining multi-environment kernels

Different multi-environment models are defined based on the construction of the kernel matrices, using information available on genotypes, molecular markers and the environment (Jarquín *et al.* 2014; Sousa *et al.* 2017; Cuevas *et al.* 2018). The construction of multi-environment kernels depends on two primary processes: the choice of covariance function and the multi-environment model.

### Choice of covariance function

Two covariance or kernel functions are generated internally; to facilitate the reading we will use the same names of the methods (GB and GK) used in Cuevas *et al.* (2016, 2017). The Genomic Best Linear Unbiased Predictor (GBLUP or **GB**) is the standard linear kernel from the properties of a multivariate normal distribution in linear mixed models and is usually referred to as the genomic relationship matrix. Thus, **GB** is obtained as follows:

$$GB=\;\frac{XX^{'}}{p}\;$$

where X is the marker matrix and p is the number of markers. This matrix was proposed by VanRaden in 2008, and since then, it has been used successfully in genomic prediction (de los Campos *et al.* 2009).

Another covariance function is the Gaussian kernel (**GK**). The **GK** appeared as a reproducing kernel (RK) in the semi-parametric model Reproducing Kernel Hilbert Spaces (RKHS) (González-Camacho *et al.* 2012) and is defined as follows:

$$GK=GK\left(x_{i},x_{j}\right)=\text{exp}\left(\frac{-hd_{ij}^{2}}{q}\right)$$

where h is the bandwidth parameter that controls the rate of decay of the covariance between genotypes, and q is the percentile of the square of the Euclidean distance $d_{ij}=\underset{k}{\sum }{\left(x_{ik}-x_{jk}\right)}^{2}$, which is a measure of the genetic distance between individuals. Results have shown that **GK** performs better than **GB** (Cuevas *et al.* 2016; Sousa *et al.* 2017). Note that the BGGE package is not limited to using the above matrices; other matrices can be used as long as they are symmetric and positive semidefinite.

### Uses of the BGGE

The BGGE package is generic and can be used to fit a great number of mixed models. For example, in genomic-enabled prediction it can be used to fit a single environment and/or multi-environments with GE including pedigree, genomic and environmental information. The conditions needed to use this first version of BGGE are: (1) must have continuous observations with multivariate normal distribution; (2) must include as many random effects as necessary, assuming they have multivariate normal distribution with variance-covariance matrices that are symmetric and positive semidefinite; (3) random errors are assumed to be homoscedastic. The main objective of this article is to describe and explain the use of the BGGE package in the context of genomic-enabled predictions. In addition, the article explains functions to generate variance-covariances of six GE models. The function used to fit these six models is considered auxiliary (because it is not the principal function).

The models considered in genomic GE were developed by Jarquín *et al.* (2014), Lopez-Cruz *et al.* (2015) and Cuevas *et al.* (2018). The six models considered in this study had a general mean μ and fixed effects $X_{f}\beta$ (for example, this could refer to the fixed effects of environments). The first multi-environment model added to μ and $X_{f}\beta$ a random vector of main genotypic effects (MM) (Jarquín *et al.* 2014), assuming these genetic effects across environments are constant, with a variance-covariance structure of $Z_{u}K{\mathrm{Z'}}_{u}^{\prime }$ (Table 1), where $Z_{u}$ is a known incidence matrix that relates the genotypes to the observations in the environments (Jarquín *et al.* 2014). The second model MM***l*** adds to the MM model a random intercept ***l*** (Table 1) with variance-covariance structure $Z_{u}I{\mathrm{Z'}}_{u}^{\prime }$ (Cuevas *et al.* 2018). The third model is the multi-environment, single variance genotype × environment deviation model (MDs), which is an extension of the main genetic effect model (MM), but incorporates a random deviation effect of GE. Table 1 shows that this component has a variance-covariance structure $\left(Z_{u}K{\mathrm{Z'}}_{u}^{\prime }\right){}^{\circ}Z_{E}{\mathrm{Z'}}_{E}$, where ° is the Hadamard product and $Z_{E}$ is a known matrix of environmental covariables (Jarquín *et al.* 2014; Sousa *et al.* 2017). When a random intercept is added to model MDs, the fourth model is MDs***l*** (Table 1). An alternative model is the multi-environment, environment-specific, variance genotype × environment deviation model (MDe) proposed by Lopez-Cruz *et al.* (2015). In MDe, a vector of specific environment effects is added with a known variance-covariance structure such that the blocks that correspond to the columns and rows of environment jth (j=(1,…,m) are a matrix $K_{j}\;$with the other elements equal to zero (Sousa *et al.* 2017). Again, when a random intercept component is added, a new model is generated, the MDe ***l*** (Cuevas *et al.* 2018).

**Table 1:** - Known variance-covariance matrices for six models of function *getGK*

Model	Main genetic effect of line across environments	Genotype × environment interaction (G×E)	Random intercept of the lines
MM	$Z_{u}K{\mathrm{Z'}}_{u}^{\prime }$		
MMl	$Z_{u}K{\mathrm{Z'}}_{u}^{\prime }$		$Z_{u}I{\mathrm{Z'}}_{u}^{\prime }$
MDs	$Z_{u}K{\mathrm{Z'}}_{u}^{\prime }$	$\left(Z_{u}K{\mathrm{Z'}}_{u}^{\prime }\right){}^{\circ}Z_{E}{\mathrm{Z'}}_{E}^{\prime }$	
MDsl	$Z_{u}K{\mathrm{Z'}}_{u}^{\prime }$	$\left(Z_{u}K{\mathrm{Z'}}_{u}^{\prime }\right){}^{\circ}Z_{E}{\mathrm{Z'}}_{E}^{\prime }$	$Z_{u}I{\mathrm{Z'}}_{u}^{\prime }$
MDe	$Z_{u}K{\mathrm{Z'}}_{u}^{\prime }$	$\left[\begin{array}{ccccc} 0 & \cdots & 0 & \cdots & 0\\ \vdots & \ddots & \vdots & \ddots & \vdots \\ 0 & \cdots & K_{j} & \cdots & 0\\ \vdots & \ddots & \vdots & \ddots & \vdots \\ 0 & \cdots & 0 & \cdots & 0 \end{array}\right]$ for each environment j (j=1,...,m)	
MDel	$Z_{u}K{\mathrm{Z'}}_{u}^{\prime }$	$\left[\begin{array}{ccccc} 0 & \cdots & 0 & \cdots & 0\\ \vdots & \ddots & \vdots & \ddots & \vdots \\ 0 & \cdots & K_{j} & \cdots & 0\\ \vdots & \ddots & \vdots & \ddots & \vdots \\ 0 & \cdots & 0 & \cdots & 0 \end{array}\right]$ for each environment j (j=1,…,m)	$Z_{u}I{\mathrm{Z'}}_{u}^{\prime }$

## Experimental Data Set

To show how to use the package, a maize data set is available that includes phenotypes, SNP markers and two kernels. The data set consists of 614 maize hybrids evaluated at Piracicaba and Anhumas, São Paulo, Brazil, in 2017. Field trials were carried out using an augmented block design, with two commercial hybrids as checks. At each site, two levels of nitrogen (N) fertilization, Ideal N (IN) and Low N (LN) were applied. The combination site and the N level formed the four environments (P-IN, P-LN, A-IN, and A-IN). The field trials carried out under ideal N conditions received 100 kg ha^-1^ of N (30 kg ha^-1^ at sowing and 70 kg ha^-1^ in a coverage application) at the V8 plant stage. The experiments carried out under low N received only 30 kg/ha of N at sowing. For each field trial, we adjusted phenotypic values by the experimental design (incomplete block). We fitted a mixed model with the random effect of the genotypes (including treatments and checks) and the random effect of the incomplete block to recover the inter block information.

The 49 parental lines were genotyped with the Affymetrix Axiom Maize Genotyping Array of 616 K SNPs (Unterseer *et al.* 2014). Quality control for call rate and missing marker imputation was applied in the parental lines. Markers with call rates lower than 0.9 and with at least one heterozygous locus were removed. Hybrid genotypes were composed by combining their respective parental lines. A second quality control was performed after a hybrid matrix was constructed, in which markers with minor allele frequency (MAF) lower than 0.05 were removed. After that, we pruned the hybrids’ SNP matrix by removing markers with a pairwise linkage disequilibrium (LD) greater than 0.9. Quality control was performed using the R package synbreed (Wimmer *et al.* 2012) and LD pruning was carried out using the SNPRelate R package (Zheng *et al.* 2012). After pre-processing the data set, 34,571 high-quality SNPs were available.

### Data and Software Availability

The BGGE R package is available at CRAN (https://cran.r-roject.org/web/packages/BGGE/BGGE.pdf). The following link hdl:11529/10548107 contains the maize data set comprising 614 maize lines under maizefiles.RData (from maizefiles.tab); ‘geno’ is the matrix of markers, ‘pheno_geno’ is the data.frame with the first column indicating the factor environment, another column corresponding to the entry’s unique ID (GID); GK denotes the Gaussian kernel matrix and GB represents the GBLUP matrix.

## Description and Application of the Bgge Package

This section shows how to use the BGGE R package, first by describing the two main principal functions in detail and then illustrating its use with a real data set. *We then show how to fit models* MM, MDs, and MDe with various kernels including GB, GK, as well as Kernel Averaging (KA).

### Describing functions

In what follows, we present the use and describe the main aspects of the two functions: *getK* and *BGGE*. The *getK* function creates multi-environment kernels or known covariance matrices for the MM, MDs, and MDe models (Sousa *et al.* 2017) with or without random intercepts MM***l***, MDs***l***, MDe***l*** (Cuevas *et al.* 2018). The objective is to help the user construct these matrices (Table 1), which will be used as entries in the *BGGE* function to be able to fit the model. Note that the use of the *BGGE* function does not depend on *getK*.

Box 1 getK(Y, X, kernel = c(“GK”, “GB”), setKernel = NULL, bandwidth = 1, model = c(“SM”, “MM”, “MDs”, “MDe”), intercept.random = FALSE, quantil = 0.5)

The *getK* function is an auxiliary function for constructing variance-covariance matrices like those shown in Table 1 using the GB (GBLUP) or Gaussian kernel (GK) methods. Box 1 (above) contains the main arguments of the *getK* function. **Y** is a data.frame phenotypic data set with three columns; the first column is a factor for environments, the second column is a factor identifying genotypes, and the third column contains the trait of interest. **X** is the marker matrix in which individuals are in rows and markers in columns, and missing markers are not allowed; the **kernel** argument is the method used to construct the GK or GB kernels. In the case of the Gaussian kernel (GK), the **bandwidth** (default is 1) and **quantile** (default is 0.5) arguments are equivalent to the bandwidth parameter and the quantile, as previously defined. The bandwidth parameter can be estimated using a Bayesian approach, as presented in Pérez-Elizalde *et al.* (2015).

When choosing a covariance matrix other than GB and GK (for example, the pedigree relationship - matrix ***A***), these kernels are passed by the **setKernel** argument (default is NULL). The argument **model** allows us to choose models MM, MDs, and MDe. Additionally, a univariate single model (SM) can be chosen. The argument **intercept.random** (default is FALSE) is an option for adding the random intercept of the genetic component (Table 1). The output of BGGE is a two-level list indicating the covariance matrix of the selected model and the type of matrix, where “D” stands for dense, and “BD” stands for block diagonal.

The main function of the package is the *BGGE* function that aims to perform genomic prediction through a linear mixed model for continuous variables.

Box 2 BGGE(y, K, XF = NULL, ne, ite = 1000, burn = 200, thin = 3)

Box 2 presents the arguments for the BGGE function. **y** is the response variable (allowing missing values). **K** is a two-level list containing the matrix (*i.e.*, **K = **list(list(Kernel = GK,Type = ”D”))) associated with each random effect vector in the model and the type of matrix (D = Dense, BD = Block diagonal). **XF** is the design matrix used to fit fixed effects, **ne** is a vector defining the number of genotypes in each environment, and **ite**, **burn**, and **thin** define the number of iterations of the sampler, the number of samples to be discarded, and the thinning used to compute posterior means, respectively. Further details on K and ne are given in the examples below.

### Example 1: fitting THE MM model

In this example, we show how to fit the main effects genotypic model (MM) (Jarquín *et al.* 2014; Sousa *et al.* 2017) along with the linear kernel GBLUP. First, we obtain the kernel through *getK*.

Box 3 rm(list = ls())

library(BGGE)

### Load the maize dataset from supplementary material

load(“maizefiles.Rdata”)

head (geno) # the marker matrix

head(pheno_geno) # the phenotypic data

K1 <- getK(Y=pheno_geno, X=geno, kernel=“GB”, model = “MM”)

The phenotypic file must be provided as a data frame with three columns that identify the environments, the individuals or genotypes, and the phenotypic observations. When in the presence of the marker matrix, it is necessary to choose the covariance function to create the kernel. The *getK* returns a two-level list with the kernels for the respective model and a definition of the type of matrix. The MM model produces only one covariance matrix (K1) considered as dense.

Box 4 ##Continue from Box 3

ne <- as.vector(table(pheno_geno$env))

fit <- BGGE(y = pheno_geno$GY, K = K1, ne = ne, verbose = T)

## K1 from Box 3

fit$yHat[pheno_geno$env == “AN_IN”] #predicted values for ##environment 1

fit$K$G$varu #genetic variance

fit$varE #residual variance

plot(fit$yHat, pheno_geno$GY)

Box 4 presents the basic syntax for the *BGGE* function. The input for K is the two-level list returned by the *getK* function. The *BGGE* function fits a multi-environment main genotypic model (MM), with a total of 1000 cycles of a Gibbs sampler (the default value for the number of iterations), and the first 200 samples are discarded (the default burn-in value). Also, samples are collected at a thinning interval of three. The *BGGE* function returns a list with estimated posterior means for each random term in the linear model and the predicted genetic values. To assess convergence and estimate the Monte Carlo error, samples of the intercept and random effect variances are stored and returned in the same output list.

### Example 2: fitting THE MDe model

In this example, we show how to fit the environment-specific variance genotype × environment deviation model (MDe) (Lopez-Cruz *et al.* 2015; Cuevas *et al.* 2016) along with the non-linear Gaussian kernel (GK).

Box 5 rm(list = ls())

library(BGGE)

### Load the maize dataset from supplementary material

load(“maizefiles.Rdata”)

ne <- as.vector(table(pheno_geno$env))

K2 <- getK(Y = pheno_geno, X=geno, kernel = “GK”, bandwidth = 1, model = “MDe”)

fit <- BGGE(y = pheno_geno$GY, K = K2, ne = ne)

fit$yHat[pheno_geno$env == “AN_LN”] #predicted values for environment 2

fit$K$G$varu #main genetic variance

fit$varE #residual variance

fit$K$AN_LN$varu #specific genetic variance

fit$varE #residual variance

plot(fit$yHat, pheno_geno$GY)

In Box 5, the *getK* function uses the Gaussian kernel and a bandwidth parameter of one and a quantile of 0.5 (default value). However, this can be modified by the bandwidth and quantile arguments. In the MDe model, the *getK* function returns, in the K2 list, the variance-covariance matrix for the main genotypic effect ($Z_{u}\mathrm{GK}{\mathrm{Z'}}_{u}^{\prime })$ (Table 1) and the kernel $\left[\begin{array}{ccccc} 0 & \cdots & 0 & \cdots & 0\\ \vdots & \ddots & \vdots & \ddots & \vdots \\ 0 & \cdots & GK_{j} & \cdots & 0\\ \vdots & \ddots & \vdots & \ddots & \vdots \\ 0 & \cdots & 0 & \cdots & 0 \end{array}\right]$ for each environment. This model is characterized by structured matrices for specific environments.

The MDe model uses the ne argument to extract the sub-matrices for each environment instead of decomposing the big sparse matrix into singular values. The BGGE returns the predicted posterior mean of genetic effects (main effect + environment-specific effects) and the estimated compound variances. Box 5 shows some elements of the output list ‘fit’, such as predictive values y in environment 2, the variance component of the main effects and the variance component specific to environment 1.

### Example 3: fitting multi-kernel multi-environment models

When using the Gaussian kernel (GK), the problem of selecting the best bandwidth parameter arises. As pointed by de los Campos *et al.* (2010), with extreme bandwidth values, the information of the markers is practically lost, making it necessary to optimize the best parameter. Endelman (2011) and Pérez-Elizalde *et al.* (2015) proposed two different approaches for optimizing this parameter via REML and the Bayesian framework, respectively. However, de los Campos *et al.* (2010) addressed the problem by proposing a multi-kernel average approach in which a sequence of kernels is obtained from a grid of bandwidth parameters, called kernel averaging (KA).

Box 6 rm(list = ls())

library(BGGE)

### Load the maize dataset from supplementary material

load(“maizefiles.Rdata”)

ne <- as.vector(table(pheno_geno$env))

K3 <- getK(Y = pheno_geno, X = X, kernel = “GK”, bandwidth = c(0.25,1,2.5), model = “MDs”)

fit <- BGGE(y = pheno_geno$GY, K = K3, ne = ne)

fit$yHat #predicted values

fit$K$G_1$varu # main genetic variance for kernel 1 (bandwidth = 0.25)

fit$K$GE_1$varu #G x E variance for kernel 1 (bandwidth = 0.25)

fit$varE #residual variance

plot(fit$yHat, pheno_geno$GY)

We use the MDs model as an example. Since the bandwidth argument accepts a vector as input, it can be used as a solution to create multi-kernels using a range of bandwidth values. For the present models, *getK* will create n×v kernels, in which n is the number of basic kernels for each model and v is the number of bandwidth parameters.

### Example 4: fitting additive + dominance models

Several kernels were proposed as *t* (Tusell *et al.* 2014) and exponential (Endelman 2011), as well as other estimators of the genomic relationship between subjects (Astle and Balding 2009; Yang *et al.* 2010; Wang and Da 2014) and the combination of non-additive kernels (Nishio and Satoh 2014) in an attempt to improve prediction. Hence, it is possible to use kernels other than GB and GK, as well as to combine them to create multi-environment kernels. In this example, we show how to apply external kernels to fit genome prediction to model MDs (Jarquín *et al.* 2014). For instance, using an SNP matrix, it is possible to compute additive and dominance relationship matrices (Azevedo *et al.* 2015) and combine them to build multi-environment kernels.

Box 7 rm(list = ls())

library(BGGE)

### Load the maize dataset from supplementary material

load(“My directory/maizefiles.Rdata”)

ne <- as.vector(table(pheno_geno$env))

ne <- as.vector(table(pheno_geno$env))

Xd <- geno

Xd[Xd == 2] <- 0

W <- (geno) #SNP matrix geno coded as 0, 1 and 2

S<-(Xd) #SNP matrix Xd coded as 0(homozygous) and 1 (heterozygous)

GBa <-tcrossprod(W)/ncol(W)#Kernel GBLUP for additive

GBd <-tcrossprod(S)/ncol(S)#Kernel GBLUP for dominance

Ker <- list(Ga = GBa, Gd = GBd)

K5 <- getK(Y = pheno_geno, setKernel = Ker, model = “MDs”)

fit <- BGGE(y = pheno_geno$GY, K = K5, ne = ne)

fit$yHat # predicted values

fit$K$G_Ga$varu #main genetic additive variance

fit$K$G_Gd$varu #main genetic dominance variance

fit$varE #residual variance

plot(fit$yHat, pheno_geno$GY)

In the initial call for *getK*, we introduce the setKernel argument that allows passing a list of kernels other than those computed internally. Thus, it creates n×k kernels, where n is the number of basic kernels for each model and k is the number of kernels introduced by the user.

### Example 5: fitting GENOMIC + PEDIGREE models

Genomic predictions can be improved by combining genomic relationship matrices and pedigree information. Legarra *et al.* (2009) proposed combining the **G** matrix and the pedigree into the **H** matrix. In contrast, Crossa *et al.* (2010) proposed that the genomic relationship and the pedigree be modeled as the sum of the two components. Hence, in this example, we show how to make predictions using genomic relationships along with pedigree information. We used the wheat data set available in BGLR (Pérez and de los Campos 2014).

Box 8 rm(list = ls())

library(BGLR)

data(wheat)

wheat.X <- scale(wheat.X)

env <- ncol(wheat.Y)

gen <- nrow(wheat.Y)

rownames(wheat.X) <- 1:gen

whe.Y <- data.frame(env = gl(n = env, k = gen),

GID = gl(n = gen, k = 1, length = gen*env),

Y = as.vector(wheat.Y))

GB <- tcrossprod(wheat.X)/ncol(wheat.X) #genomic relationship

Kga <- list(G = list(Kernel = GB, Type = “D”),

A = list(Kernel = wheat.A, Type = ”D”))

y <- whe.Y[whe.Y$env == 1, 3]

fit <- BGGE(y = y, K = Kga, ne = 599)

fit$yHat # predicted values

fit$K$G$varu #genetic additive variance (markers)

fit$K$A$varu #genetic additive variance (pedigree)

fit$varE #residual variance

plot(fit$yHat, y)

In Box 8, we fit the genomic + pedigree model for environment 1. To do this, we combined the genomic matrix and the pedigree in a list. In the list used as input for BGGE, the type of matrices is assigned as dense. For the BGGE function, since there is only one environment, ne is the number of genotypes evaluated in environment 1.

### Empirical comparisons

The method applied in BGGE using different features was compared to the standard Bayesian kernel regression proposed by de los Campos *et al.* (2010) (BGLR). The comparison of the performance of methods BGGE and BGLR was based on: (i) comparing their variance components, and (ii) comparing the computing time to the time it takes to fit three different genotype × environment models. The posterior variance components were estimated using full data. The computational time was also included in the comparison. The genomic GE models were fitted using BGLR (Pérez and de los Campos 2014) through the RKHS model and BGGE packages, using a Gibbs sampler with 60,000 iterations, a burn-in of 10,000 and a thinning interval of 10, with 5,000 samples for inference at the end. Kernels for GE models were built into the *getK* function.

The approach used for prediction includes an orthogonal transformation of the model. Despite the expected theoretical difference between these two approaches, the observed difference was not significant. For the two data sets, the residual variance was slightly lower when using the BGGE approach (Table 2). In contrast, the genetic variance components were high for BGGE. Despite this, there is no clear advantage in using one package instead of the other. However, computational time of the BGGE was up to five times faster than that of the BGLR approach (Table 3). The BGLR package uses approaches to fit generalized linear models and thus fits a wide range of Bayesian regression models like Bayesian LASSO, Bayes A, and Bayes B, among others. The BGGE package specializes in linear mixed models with some features for GE kernels.

**Table 2:** - HEL data set. Estimates of variance components obtained by the BGGE and BGLR functions for the multi-environment models, main genotypic effect model (MM), single variance G×E deviation model (MDs) and the environment-specific variance G×E deviation model (MDe) with a G-BLUP kernel

Factor	BGGE			BGLR		
	MM	MDs	MDe	MM	MDs	MDe
$\sigma^{2}$	0.749 (0.02)	0.737 (0.02)	0.733 (0.02)	0.75 (0.02)	0.736 (0.02)	0.739 (0.02)
$\sigma_{u}^{2}$	0.335 (0.08)	0.331 (0.08)	0.335 (0.08)	0.278 (0.06)	0.271 (0.06)	0.273 (0.06)
$\sigma_{uE}^{2}$	—	0.019 (0.009)	—	—	0.021 (0.007)	—
$\sigma_{PI\_LN}^{2}$	—	—	0.028 (0.02)	—	—	0.015 (0.01)
$\sigma_{PI\_IN}^{2}$	—	—	0.022 (0.02)	—	—	0.014 (0.008)
$\sigma_{AN\_LN}^{2}$	—	—	0.029 (0.02)	—	—	0.014 (0.008)
$\sigma_{AN\_IN}^{2}$	—	—	0.052 (0.03)	—	—	0.021 (0.01)

**Table 3:** - Total time (in seconds) to execute the BGGE and BGLR functions for the multi-environment models, main genotypic effect model (MM), single variance G×E deviation model (MDs) and environment-specific variance G×E deviation model (MDe) with the G-BLUP kernel

Model	BGGE	BGLR
MM	103.16	249.06
MDs	183.43	709.74
MDe	219.03	1142.73

The main mechanisms that increase the speed of the process for fitting the models are: the reparameterization of the model and the way sparse block diagonal matrices are handled. In the context of genomic parametric regression, Cuevas *et al.* (2014) showed that the new parameterization allows reducing the dimensionality; moreover, it gives a computational advantage because it allows simulations with univariate distributions for a smaller number of parameters. The extra features of the sparse structure matrix assumed in the BGGE algorithm reduce dimensionality by decreasing the computational time.

### Conclusions

The proposed package was built to make genomic predictions for continuous variables focused on genomic GE models. Using information from multi-environment trials can improve prediction, and several models have been created (Sousa *et al.* 2017; Cuevas *et al.* 2018). However, each GE model has its own properties and, therefore, specific kernels must be created in the BGGE.

The purpose of the *getK* is to generate kernels for six genomic GE models. Hence, multi-environment kernels are produced using covariance functions created internally (GB or GK). Also, there is an extra argument that allows other kernels to be passed, which opens the possibility of combining different kernels, such as additive with dominance or pedigree, for multi-environment models. For the Gaussian kernel, different values of bandwidth parameters can be introduced to create several kernels, as defined in kernel averaging (de los Campos *et al.* 2010). The output produced by *getK* is in the proper format to be used in the BGGE prediction function.

The BGGE function uses a reparametrization (Cuevas *et al.* 2014) of the linear mixed model regression in the Bayesian context. These features allow simulations with univariate distributions. We also explored the properties of structured sparsity in some GE kernels to decrease the computational time. Therefore, the package is a fast and efficient option for predicting genetic values. The BGGE was programmed entirely in R and does not have dependencies.

## Appendix

The conditional posterior distribution of b:

$$p\left(b|d,S,\sigma_{u}^{2},\tau \;\right)=\overset{n}{\underset{i=1}{\prod }}N\left(b_{i}|\frac{\tau \;\sigma_{u}^{2}s_{i}d_{i}}{1+\tau \;\sigma_{u}^{2}s_{i}},\frac{s_{i}\sigma_{u}^{2}}{1+\tau \sigma_{u}^{2}s_{i}}\right)$$

where $\tau =1/\sigma_{\varepsilon }^{2}$. Assuming that b=U'u, the genetic effects u can be recovered by u=Ub.

The conditional distributions of $\sigma_{\varepsilon }^{2}$, $\sigma_{u}^{2}$, $Sc_{u}$ and $Sc_{\varepsilon }$ are:

$$p\left(\sigma_{\varepsilon }^{2}|y^{*},u,\nu_{\varepsilon },Sc_{\varepsilon }\right)=\chi^{-2}\left(\sigma_{\varepsilon }^{2}|n+\nu_{\varepsilon },(y^{*}-u)'(y^{*}-u)+Sc_{\varepsilon }\right)$$

$$p\left(\sigma_{u}^{2}|b,\nu_{u},Sc_{u}\right)=IG\left(\sigma_{u}^{2}|\frac{\nu_{u}+n}{2},\frac{\mathrm{b'}S^{-1}b+\nu_{u}Sc_{u}}{2}\right)$$

$$Sc_{\varepsilon }=var(y)(\nu_{\varepsilon }+2)(1-R2)$$

$$Sc_{u}=\;var(y)(\nu_{\varepsilon }+2)(R2)/mean(diag\left(K_{r}\right))$$

where R2 is the proportion of variance that one expects, a priori, to be explained by the regression, with a default value of 0.5.
